# Epigenetic alterations of TP53INP1 by EHMT2 regulate the cell cycle in gastric cancer

**DOI:** 10.1186/s40164-024-00554-y

**Published:** 2024-08-19

**Authors:** Tae Young Ryu, In Hwan Tae, Tae-Su Han, Jinkwon Lee, Kwangho Kim, Yunsang Kang, Solbi Kim, Hyo Jin Lee, Cho-Rok Jung, Jung Hwa Lim, Dae-Soo Kim, Mi-Young Son, Hyun-Soo Cho

**Affiliations:** 1 Korea Research Institute of Bioscience and Biotechnology, Daejeon, 34141 Republic of Korea; 2 Korea University of Science and Technology, Daejeon, 34316 Republic of Korea; 3 Chungnam National University College of Medicine, Daejeon, 35015 Republic of Korea; 4 Department of Biological Science, Sungkyunkwan University, Suwon, 16419 Republic of Korea

**Keywords:** EHMT2, Gastric cancer, Cell cycle, TP53INP1

## Abstract

**Background:**

Gastric cancer (GC) is a type of cancer with high incidence and mortality rates. Although various chemical interventions are being developed to treat gastric cancer, there is a constant demand for research into new GC treatment targets and modes of action (MOAs) because of the low effectiveness and side effects of current treatments.

**Methods:**

Using the TCGA data portal, we identified EHMT2 overexpression in GC samples. Using RNA-seq and EHMT2-specific siRNA, we investigated the role of EHMT2 in GC cell proliferation and validated its function with two EHMT2-specific inhibitors. Through the application of 3D spheroid culture, patient-derived gastric cancer organoids (PDOs), and an in vivo model, we confirmed the role of EHMT2 in GC cell proliferation.

**Results:**

In this study, we found that EHMT2, a histone 3 lysine 9 (H3K9) methyltransferase, is significantly overexpressed in GC patients compared with healthy individuals. Knockdown of EHMT2 with siRNA induced G1 cell cycle arrest and attenuated GC cell proliferation. Furthermore, we confirmed that TP53INP1 induction by EHMT2 knockdown induced cell cycle arrest and inhibited GC cell proliferation. Moreover, specific EHMT2 inhibitors, BIX01294 and UNC0638, induced cell cycle arrest in GC cell lines through TP53INP1 upregulation. The efficacy of EHMT2 inhibition was further confirmed in a 3D spheroid culture system, PDOs, and a xenograft model.

**Conclusions:**

Our findings suggest that EHMT2 is an attractive therapeutic target for GC treatment.

**Supplementary Information:**

The online version contains supplementary material available at 10.1186/s40164-024-00554-y.

## To the editor

Gastric cancer (GC) is the fifth most prevalent cancer globally and the fourth leading cause of cancer-related death [1]. Chemotherapy, including 5-fluorouracil, cisplatin, and doxorubicin, is an important strategy for the treatment of gastric cancer (GC) and can be used before, after, or during surgery [2, 3]. However, due to the high side effects and low effectiveness of current treatments, there is a growing demand for the development of new therapeutic targets for gastric cancer, thus it is crucial to elucidate the molecular mechanisms of GC progression and identify effective therapeutic targets for treatment.

**Fig. 1:**
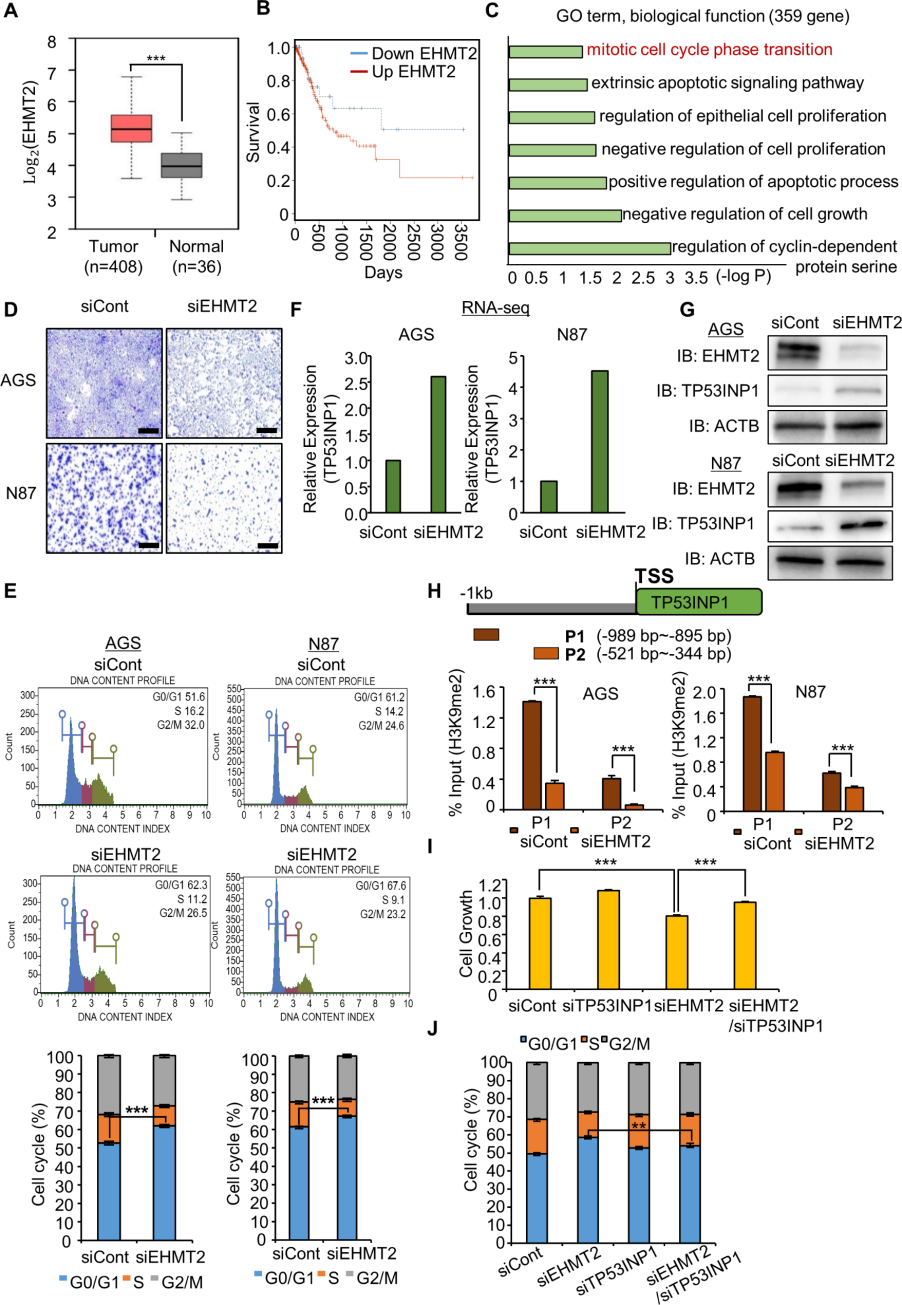
EHMT2 knockdown suppresses the growth of AGS and NCI-N87 cells by inducing cell cycle arrest. **A** EHMT2 expression in normal and GC samples derived from the TCGA portal. All p values were calculated via Student’s t test (***p < 0.001). **B** Kaplan‒Meier plot of EHMT2 in GC samples. **C** DAVID-based GO analysis of the RNA-seq results for the 359 upregulated genes. The enriched terms are shown. **D** Cell growth assay after treatment with siEHMT2 and siCont for 48 h. AGS and NCI-N87 cells were fixed in 100% methanol and stained with crystal violet solution. Scale bar, 500 μm. **E** FACS analysis via PI staining was performed after the treatment of AGS and NCI-N87 cells with siEHMT2. All p values were calculated via Student’s t test (***p < 0.001). **F** Expression level of TP53INP1 by RNA-seq after treatment with siEHMT2. **G** Western blot analysis after EHMT2 knockdown via anti-EHMT2 and TP53INP1 antibodies. **H** Graphical abstract of the ChIP primer design for the TP53INP1 promoter region (upper). The ChIP assay was performed using an anti-H3K9me2 antibody. The results are expressed as a percentage of input chromatin compared with the control in AGS and NCI-N87 cells after siEHMT2 treatment. The means ± SDs of three independent experiments are shown. All p values were calculated via Student’s t tests (***p < 0.001) (lower). **I** CCK-8 solution was added to the culture medium, and the cells were incubated for 5 min at 37 °C. The intensity of cell growth was measured via a microplate reader (450 nm). The means ± SDs of three independent experiments are shown. All p values were calculated via Student’s t test (***p < 0.001). **J** FACS analysis via PI staining was performed after the cotransfection of AGS and NCI-N87 cells with siEHMT2 and siTP53INP1. All p values were calculated via Student’s t test (**p < 0.01)

Here, we demonstrated using RNA-seq results derived from TCGA portal that euchromatic histone lysine methyltransferase 2 (EHMT2) is significantly overexpressed in GC patients (*n* = 408) compared with normal individuals (*n* = 36) and that this phenotype is associated with a poorer prognosis (Fig. 1A and B). EHMT2, also known as G9a, is a histone methyltransferase that regulates the mono- and di-methylation of H3K9 [4, 5]. In the Gene Ontology (GO) analysis with differentially expressed genes (359 upregulated genes and 347 downregulated genes) of the RNA-seq results, cell growth and cell cycle-related terms were significantly enriched in the EHMT2 knockdown group (Fig. 1C, Supplemental Fig. 1A and B). Moreover, we observed a reduction in cell growth (Fig. 1D, Supplemental Fig. 1C), Ki-67 intensity (Supplemental Fig. 1D), and G1 arrest (Fig. 1E) after the knockdown of EHMT2. Next, we assessed cell cycle arrest mediated by EHMT2 in more detail, and we confirmed that these phenomena were induced by the epigenetic regulation of the tumor protein p53-inducible nuclear protein 1 (TP53INP1) [6, 7] by EHMT2. RNA-seq and Western blot analysis revealed that the expression level of TP53INP1 was increased by EHMT2 knockdown (Fig. 1F and G, Supplemental Fig. 2A). The H3K9 dimethylation level at the promoter region of TP53INP1 was decreased in the siEHMT2 group (Fig. 1H). Next, we carried out a recovery cell growth assay using siEHMT2 and siTP53INP1 to identify TP53INP1-related antiproliferative effects. After cotreatment with siEHMT2 and siTP53INP1, the growth inhibition and G1 arrest caused by EHMT2 knockdown were restored in the siEHMT2 and siTP53INP1 cotreatment group (Fig. 1I and J, Supplemental Fig. 2B and C). Thus, EHMT2 directly regulates TP53INP1 to promote GC cell proliferation.

**Fig. 2:**
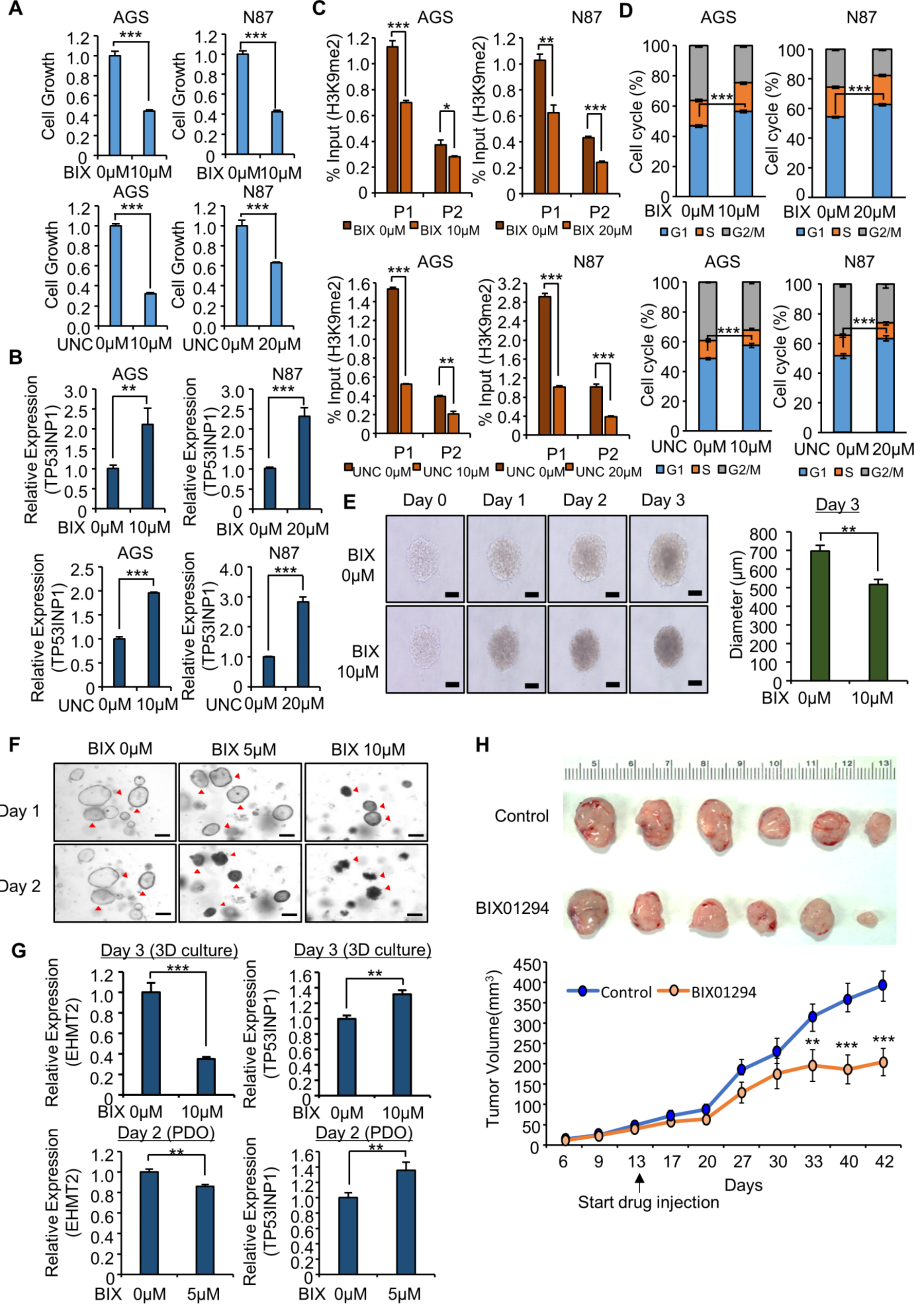
BIX01294 and UNC0638, specific EHMT2 inhibitors, suppress the growth of AGS and NCI-N87 cells. **A** For the cell viability assay, the cells were incubated for 5 min at 37 °C after the addition of CCK-8 solution. The intensity of cell growth was measured via a microplate reader (450 nm). The means ± SDs of three independent experiments are shown. All p values were calculated via Student’s t test (***p < 0.001). **B** qRT‒PCR analysis of EHMT2 and TP53INP1 after treatment with BIX01294 or UNC0638. All p values were calculated via Student’s t test (**p < 0.01, ***p < 0.001). **C** The ChIP assay was performed using an anti-H3K9me2 antibody. The results are expressed as the percentage of input chromatin compared with the control in AGS and NCI-N87 cells after BIX01294 or UNC0638 treatment. The means ± SDs of three independent experiments are shown. All p values were calculated via Student’s t tests (*p < 0.05, **p < 0.01, ***p < 0.001). **D** FACS analysis via PI staining was performed after the treatment of AGS and NCI-N87 cells with BIX01294 (upper) or UNC0638 (lower). P values were calculated via Student’s t test (***p < 0.001). **E** 3D spheroid formation assay with the NCI-N87 cell line. The cells were photographed under a microscope each day. Scale bar, 500 μm (left). The size of the spheroids was measured via ImageJ software (right). The means ± SDs of three independent experiments are shown. All p values were calculated via Student’s t tests (**p < 0.01). **F** Representative images of GC organoids treated with BIX01294. Scale bar: 500 μm. **G** qRT‒PCR analysis of EHMT2 and TP53INP1 after treatment with BIX01294. All p values were calculated via Student’s t test (**p < 0.01, ***p < 0.001) (upper; 3D culture), (lower; PDO). **H** BIX01294 treatment suppressed the growth of xenograft nude mouse tumors. Macroscopic image of tumors on day 42 (upper) and tumor volume (lower) [p values were calculated via 2-way ANOVA (***, *P* < 0.001)]

BIX01294 and UNC0638 are specific inhibitors that decrease the activity of EHMT2 [8, 9]. After the AGS and NCI-N87 cell lines were treated with either BIX01294 or UNC0638, we observed a reduction in cell proliferation (Fig. 2A, Supplemental Fig. 3A) and upregulation of TP53INP1 gene and protein expression (Fig. 2B, Supplemental Fig. 3B). In the ChIP assay, after treatment with BIX01294 or UNC0638, the H3K9 dimethylation status in the promoter region of TP53INP1 decreased, consistent with the results of siEHMT2 treatment (Fig. 2C). Moreover, G1 arrest and a reduction in Ki-67 staining were observed after BIX01294 or UNC0638 treatment (Fig. 2D, Supplemental Fig. 3C and D). Finally, to investigate EHMT2 as a potential therapeutic target for GC, we examined this effect in a 3D culture system and in patient-derived cancer organoids (PDOs). We observed a decrease in the size of the 3D spheroids, confirming that BIX01294 also inhibits GC growth in a 3D spheroid culture system (Fig. 2E). We confirmed that the size of the patient-derived gastric organoids decreased (Fig. 2F). qRT‒PCR analysis revealed that the expression of TP53INP1 increased in response to BIX01294 treatment in both the 3D culture system and the PDO system (Fig. 2G). Finally, to confirm the inhibitory effect of BIX01294 on tumor growth in vivo, we established a subcutaneous mouse model. The xenograft results revealed that the group treated with BIX01294 presented decreases in tumor size and weight (Fig. 2H, Supplemental Fig. 4A). Thus, we suggest that EHMT2 inhibition can effectively inhibit GC growth and propose that EHMT2 is an attractive therapeutic target for GC (Supplemental Fig. 4B).

In this study, we analyzed RNA-seq data. Considering that EHMT2 is responsible for H3K9 dimethylation, we selected upregulated genes from the RNA-seq data after siEHMT2 treatment as primary targets [10]. After genes related to the cell cycle were assessed, TP53INP1 was ultimately chosen as a potential target of EHMT2. The candidate approach to target selection certainly has limitations. EHMT2 indeed regulates the expression of TP53INP1, but it is likely to also regulate other cell cycle-related genes. In future studies, performing H3K9 dimethylation ChIP-seq could enable a more accurate analysis of EHMT2-mediated cell cycle regulation. Here, we employed a specific inhibitor of EHMT2, BIX01294, to investigate the function of EHMT2. However, as shown in Fig. 2G, treatment with BIX01294 resulted in decreased expression of EHMT2 in the 3D spheroid and PDO system. While BIX01294 can increase the expression of TP53INP1 by reducing the activity of EHMT2, we also anticipate that BIX01294 might regulate the expression of EHMT2 through nonspecific effects. Thus, the development of EHMT2-specific inhibitors with reduced side effects will be an important step in the development of treatments for gastric cancer.

In conclusion, EHMT2 has emerged as a promising therapeutic target for various cancer types, including GC, suggesting that the development of EHMT2-specific inhibitors could pave the way for effective cancer treatment.

### Electronic supplementary material

Below is the link to the electronic supplementary material.

Supplementary Material 1

## Data Availability

No datasets were generated or analysed during the current study.

## Abbreviations

ACK: Ammonium chloride potassium
BIX: BIX01294
ChIP: Chromatin immunoprecipitation
CCK-8: Cell Counting Kit-8
DAVID: Database for annotation, visualization and integrated discovery
DEG: Differentially expressed gene
DMSO: Dimethyl sulfoxide
ECL: Enhanced chemiluminescence
EHMT2: Euchromatic histone-lysine N-methyltransferase 2
FACS: Fluorescence-activated cell sorting
FBS: Fetal bovine serum
FPKM: Fragments per kilobase of transcripts per million mapped reads
GC: Gastric cancer
GO: Gene ontology
H3K9: Histone 3 lysine 9
H&E: Hematoxylin and eosin
KEGG: Kyoto Encyclopedia of Genes and Genomes
Ki-67: Antigen Kiel 67
PBS: Phosphate-buffered saline
PDO: Patient-derived organoid
PI: Propidium iodide
RNA-seq: RNA sequencing
siRNA: Small interfering RNA
TCGA: The Cancer Genome Atlas
TP53INP1: Tumor protein p53 inducible nuclear protein 1
UNC: UNC0638
