# Supplementary figures and images for: Epigenetic alterations of TP53INP1 by EHMT2 regulate the cell cycle in gastric cancer
Source: Exp Hematol Oncol. 2024 Aug 19;13:86. doi: 10.1186/s40164-024-00554-y (PMC11334499; doi:10.1186/s40164-024-00554-y)

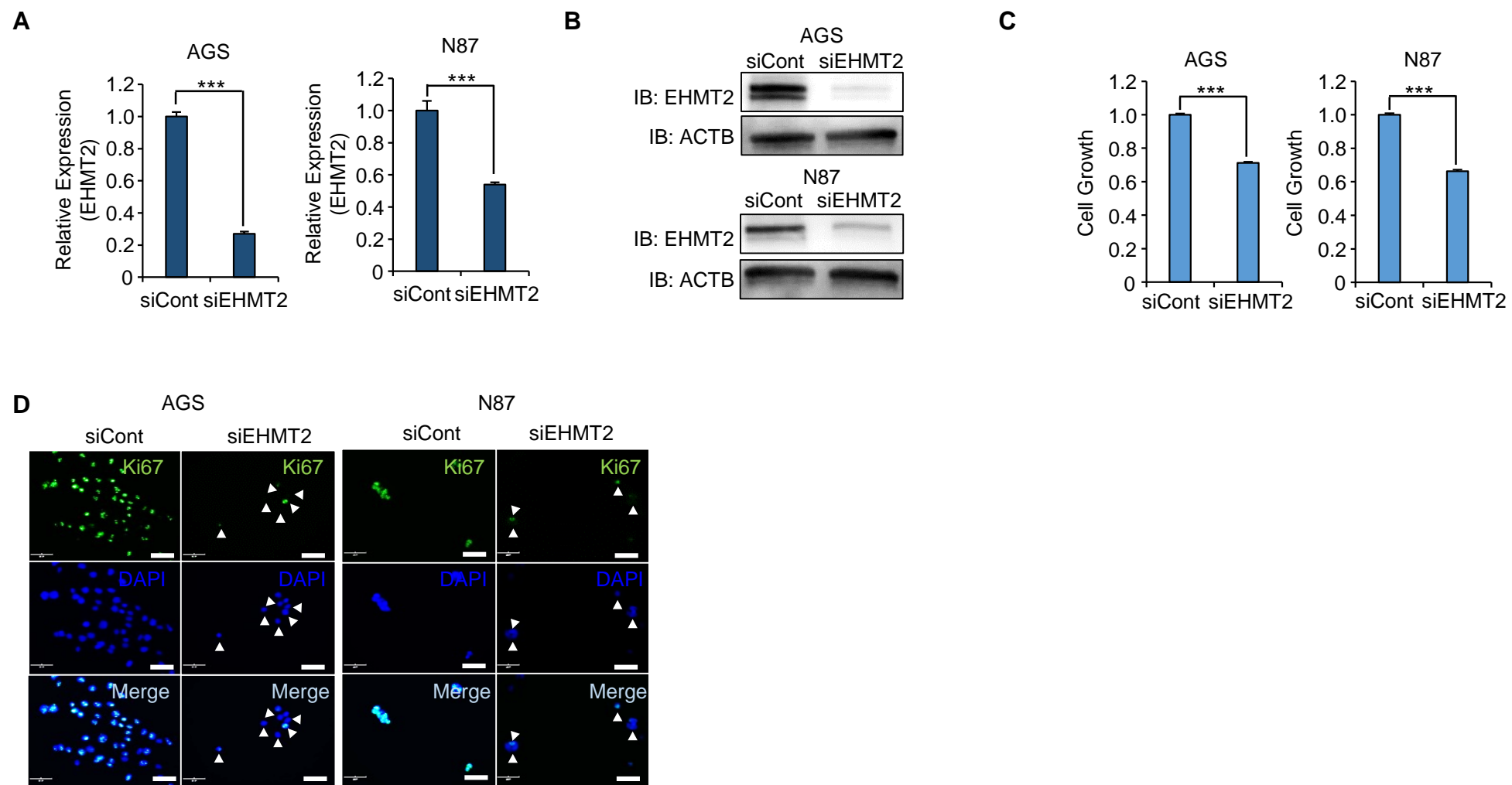

Supplemental Fig. 1

**A**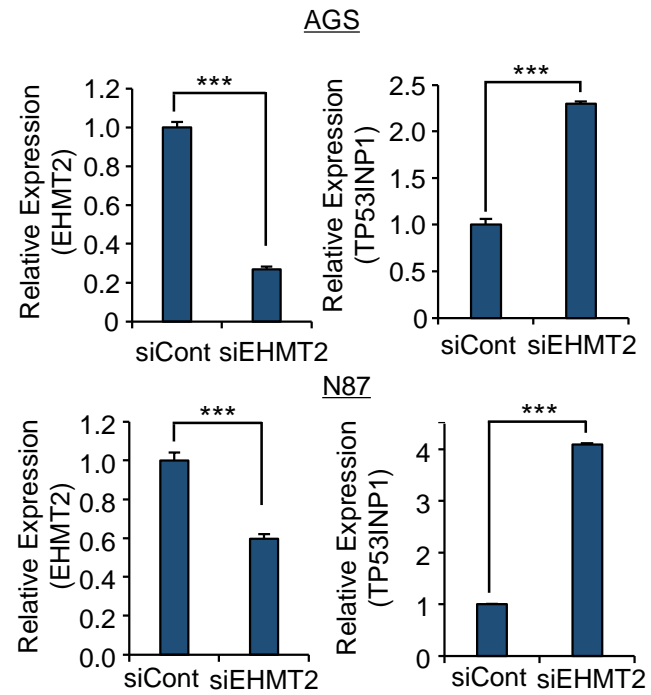**B**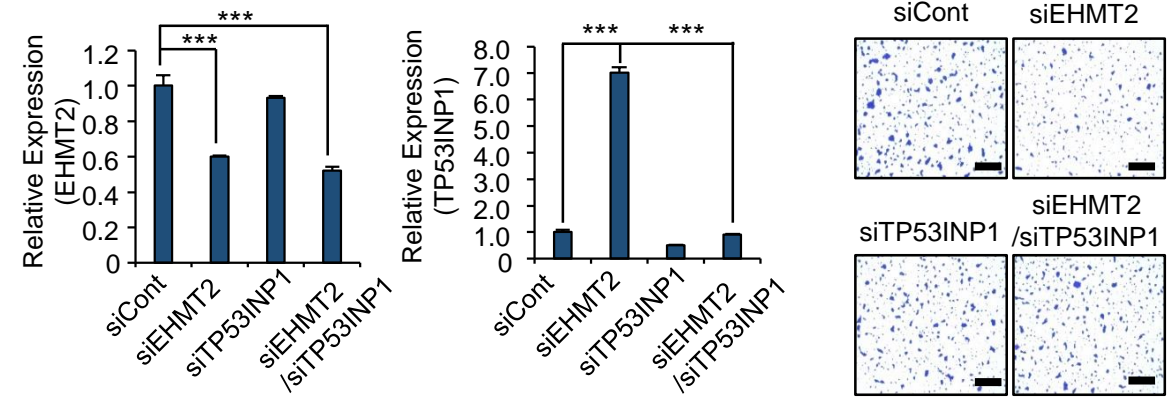**C**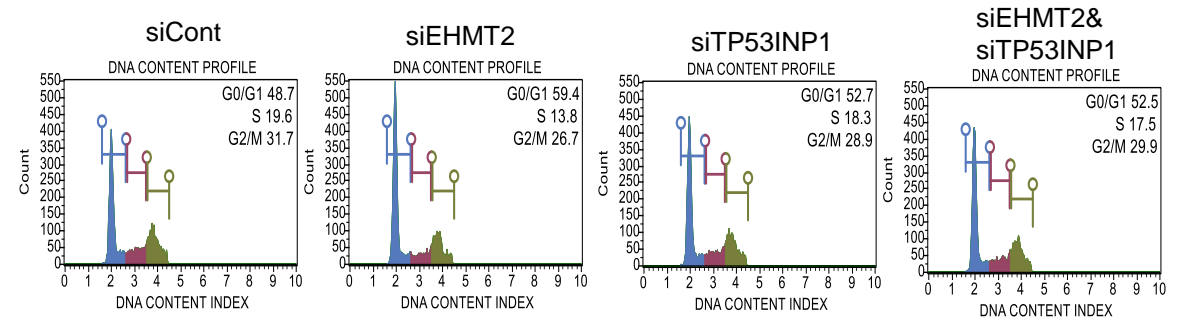

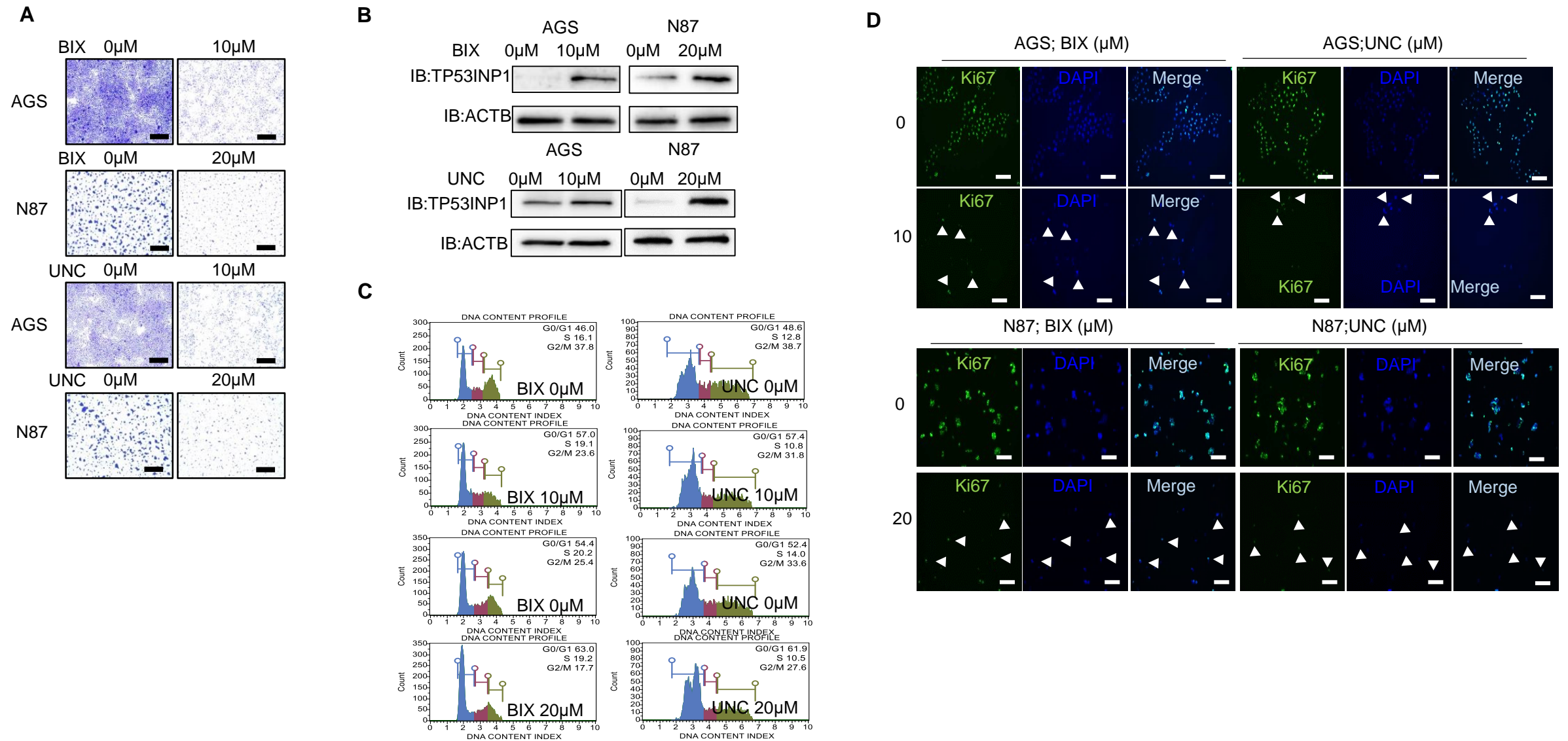

Supplemental Fig. 3

**A**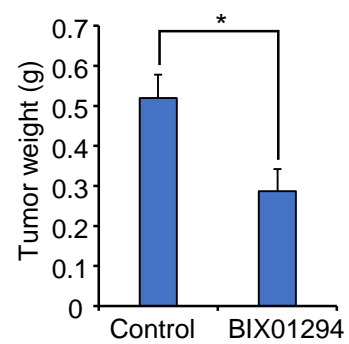**B**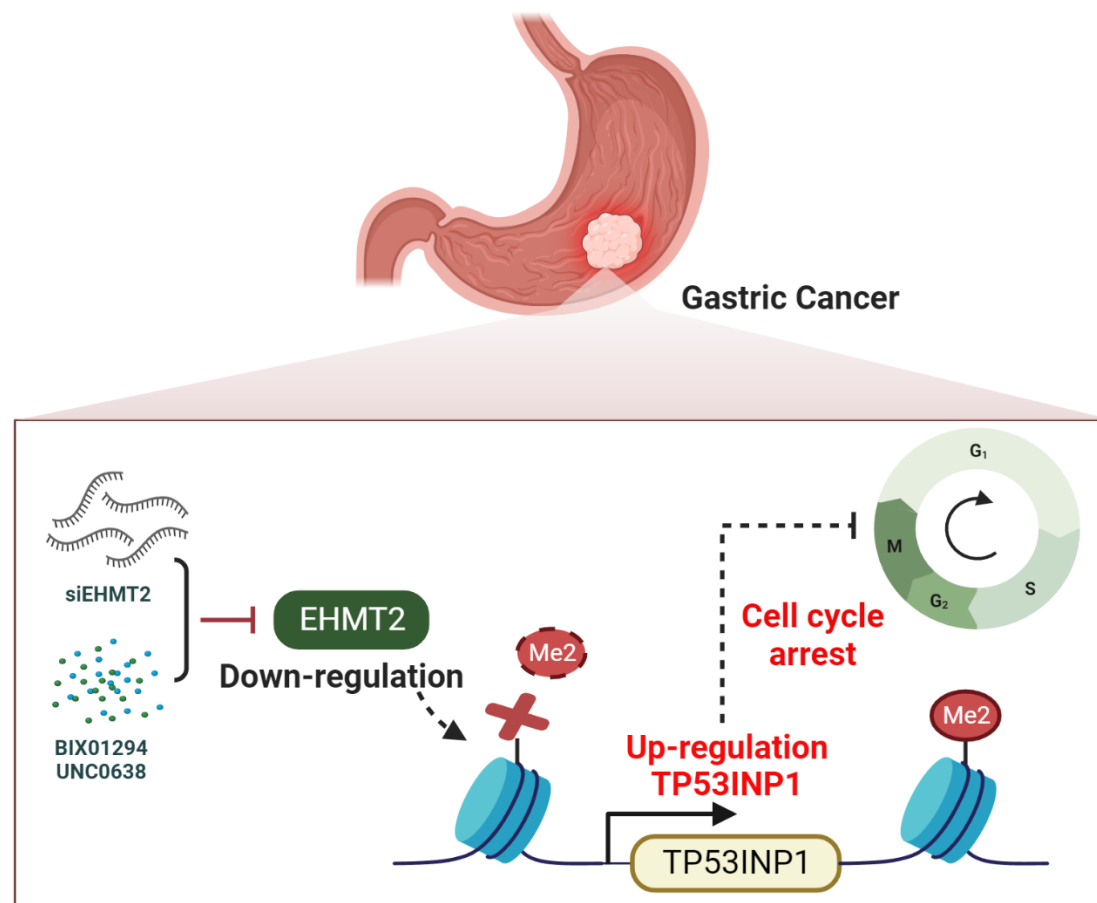

Supplement: Supplementary file 1 — Supplementary Material 1 [file 40164_2024_554_MOESM1_ESM.pdf]
